# Neuroimaging in aging: brain maintenance

**DOI:** 10.12688/f1000research.11419.1

**Published:** 2017-07-25

**Authors:** Lars Nyberg

**Affiliations:** 1 Umeå Center for Functional Brain Imaging (UFBI), Umeå University, Umeå, Sweden; 2 Department of Integrative Medical Biology, Umeå University, Umeå, Sweden; 3 Department of Radiation Sciences, Umeå University, Umeå, Sweden

**Keywords:** multimodal neuroimaging, aging, brain maintenance

## Abstract

Neuroimaging studies of the aging brain provide support that the strongest predictor of preserved memory and cognition in older age is brain maintenance, or relative lack of brain pathology. Evidence for brain maintenance comes from different levels of examination, but up to now relatively few studies have used a longitudinal design. Examining factors that promote brain maintenance in aging is a critical task for the future and may be combined with the use of new techniques for multimodal imaging.

## Introduction

Modern neuroimaging techniques increasingly are being used to understand the brain bases of cognitive impairment in aging and also to investigate what characterizes the brains of older adults with relatively spared functioning. With a growing elderly population worldwide, these issues are of great significance and the field of cognitive neuroscience of aging is rapidly expanding ^1^. At the same time, the neuroimaging field in general is characterized by many challenges ^2^, and there is marked conceptual, terminological, and methodological diversity in various applications of neuroimaging techniques to study the aging brain. Here, I will summarize recent findings that support the notion that the best neuroimaging predictor of preserved memory and cognition in older age is brain maintenance ^3^.

## Capturing individual differences

Many kinds of memory and cognition decline in aging, but it is important to stress that there are marked individual differences in onset and rate of change ^4^. One important concept for understanding this kind of heterogeneity is that of cognitive reserve, which has been defined as follows: “individual differences in how people process tasks allow some to cope better than others with brain pathology” ^5^. More recently, investigators introduced the concept of brain maintenance, which can be defined as follows: “individual differences in the manifestation of age-related brain changes and pathology allow some people to show little or no age-related decline” ^3^. Thus, the maintenance concept departs from the fact that, just as there is marked variability in cognition within the older population, there are marked individual differences in the extent of brain changes in aging.

## Brain-cognition link

A key prediction of the brain-maintenance account is that older adults with relatively well-preserved memory and cognition should have few brain changes. Several studies using different neuroimaging methods have provided support for this prediction. These include magnetic resonance imaging (MRI) studies of hippocampal volume in relation to episodic memory, MRI studies of cortical thickness in relation to executive functions, MRI studies of white matter connectivity in relation to working memory, functional MRI (fMRI) studies of episodic and working memory, positron emission tomography (PET) studies of dopamine D1 and D2 systems in relation to interference resolution, and PET studies of amyloid burden in relation to episodic and working memory (reviewed in 3). Additional evidence is provided by more recent studies. An fMRI study used episodic-memory longitudinal data acquired prior to the imaging session to classify participants into average and successful older adults ^6^. When the two groups were compared regarding brain-activity patterns during an episodic-memory encoding task, it was found that the successful older adults showed higher hippocampus activation than the average group. In other words, maintained functionality of the hippocampus was related to preserved memory. Relatedly, a study found that older adults whose activation pattern deviated less from the average pattern of younger adults during an associative cognitive task had higher overall memory and lower levels of false recognition ^7^. A final example is provided by the results of a recent PET study of tau deposition, in which it was found that increased tau tracer retention in the medial temporal lobe predicted worse episodic-memory performance ^8^.

## Imaging of change

The notion of brain maintenance receives much support from many kinds of studies, but to date most have used a cross-sectional design comparing separate groups of younger and older adults. By contrast, relatively few neuroimaging studies have used a longitudinal design to map within-person changes in brain and cognition and in particular to examine how the two variables were inter-related in so-called change-change analyses. This is a noteworthy omission as it is generally agreed that the longitudinal design offers stronger support for conclusions on change. However, the findings from two recent longitudinal neuroimaging studies provide support for brain maintenance. In one structural MRI study, 15-year changes in episodic memory, word fluency, fluid IQ, and processing speed were related to 4-year changes in cortical and subcortical gray matter volume and white matter connectivity and lesions ^9^. Of the many examined brain and cognitive parameters, only age-related hippocampal atrophy was significantly related to memory change. It was concluded that medial-temporal lobe integrity is crucial for the maintenance of episodic-memory functioning in older age. The other MRI study examined longitudinal changes in functional connectivity at resting state and related these changes to 15-year changes in episodic memory ^10^. It was found that over time an elevation of functional connectivity in the posterior medial-temporal cortex was associated with decreasing memory. It should be stressed that no such relation was seen in the anterior medial-temporal lobe, and this is in line with the notion of functional heterogeneity along the hippocampus longitudinal axis and among different hippocampus subfields, as reviewed elsewhere ^11, 12^.

## Multimodal neuroimaging

The neuroimaging field is characterized by rapid methods development, in terms of both analyses and data acquisition. Today, it is not uncommon for a single study to include both advanced MRI and PET methodologies. A recent example is a large-scale study of 181 adults between 64 and 68 years of age, in which PET was used to map the dopamine D2 system and MRI to measure brain activity, gray and white matter structure, and perfusion ^13^. An extensive cognitive battery was also included, and it was found that caudate D2 was related to episodic memory as well as to functional connectivity between the ventral striatum and medial temporal cortex. Given the use of a narrow age cohort design, the study did not provide any information of direct relevance to aging, but a longitudinal follow-up is planned ^14^. A promising neuroimaging application for the future is hybrid/simultaneous PET-MRI ^15^, which facilitates the integration of various kinds of imaging data. One critical issue that multimodal imaging can shed light on is the specificity versus inter-relatedness of various brain measures in accounting for individual differences in cognitive change (see the chapter by Fjell and Walhovd in 1).

## Conclusions

Neuroimaging studies in aging have begun to reveal the brain bases for preserved memory and cognition in older age. Findings from different levels of investigation (such as neurochemical, gray and white matter integrity, and systems-level activation patterns) converge to support the notion of brain maintenance, such that relative lack of brain pathology constitutes the primary determinant of successful cognitive aging. For the future, further examination of the factors that promote brain maintenance in aging is critical as it may inform attempts at prevention and intervention. These factors likely include lifestyle factors, such as exercise and diet ^16^.

## Abbreviations

D, dopamine; fMRI, functional magnetic resonance imaging; MRI, magnetic resonance imaging; PET, positron emission tomography.
